# Efficacy of Oral Medications or Intrauterine Device-Delivered Progestin in Patients with Endometrial Hyperplasia with or without Atypia: A Network Meta-Analysis

**DOI:** 10.3390/jcm12082980

**Published:** 2023-04-19

**Authors:** Yu-Fei Zhang, Yu Fan, Yi Mu, Jin-Ke Li

**Affiliations:** 1 Department of Gynecology and Obstetrics, West China Second Hospital, Sichuan University, Chengdu 610041, China; 2 Key Laboratory of Birth Defects and Related Diseases of Women and Children of Ministry of Education, Sichuan University, Chengdu 610041, China

**Keywords:** endometrial hyperplasia, hormonal therapy, levonorgestrel-releasing intrauterine system, oral progestin, network meta-analysis

## Abstract

The aim of this systematic review was to evaluate the efficacy of oral medication or intrauterine device-delivered progestins in patients with endometrial hyperplasia (EH) with or without atypia. We systematically examined PubMed, EMBASE, the Cochrane Library, and clinicaltrials.gov to identify studies reporting the regression rate of patients with EH who received progestins or non-progestins. The regression rates after different treatments were compared using a network meta-analysis in terms of the relative ratios (RRs) and 95% confidence intervals (CIs). Begg–Mazumdar rank correlation and funnel plots were performed to evaluate the publication bias. Five non-randomized studies and 21 randomized controlled trials involving 2268 patients were included in the network meta-analysis. The levonorgestrel-releasing intrauterine system (LNG-IUS) was associated with a higher regression rate than medroxyprogesterone acetate (MPA) (RR 1.30, 95% CI 1.16–1.46) in patients with EH. Among those without atypia, the LNG-IUS was associated with a higher regression rate than any of the three types of oral medications (MPA, norethisterone, or dydrogesterone (DGT)) (RR 1.35, 95% CI 1.18–1.55). According to the network meta-analysis, combining the LNG-IUS with MPA or metformin increased regression rate, while DGT was associated with the highest regression rate among all oral medications. The LNG-IUS may be the best choice for patients with EH, and combining it with MPA or metformin may further improve its efficacy. DGT may be the preferred choice for patients who are unwilling to use the LNG-IUS or who cannot tolerate its side effects.

## 1. Introduction

Endometrial hyperplasia (EH) is a non-invasive, abnormal proliferation of endometrial glands or stroma of the uterus, and it increases the risk of endometrial cancer [1]. The World Health Organization defines two types of EH, with or without atypia [2]. The type with atypia is thought to be highly precancerous, with the condition becoming malignant in almost 60% of cases within five years of diagnosis [3]. Both types rarely occur in women younger than 30 years; the type with atypia is diagnosed most often in women 60–64 years old, and the type without atypia in women 50–54 years old [4]. Among premenopausal women, the incidence rate of EH with atypia is 7 per 100,000 woman-years, while that of EH without atypia is 30 per 100,000 woman-years [5].

The most common symptom of EH is abnormal uterine bleeding, which means more frequent or severe bleeding in the case of premenopausal women or any uterine bleeding in the case of postmenopausal women [6]. A systematic review of studies on premenopausal women concluded that the risk of endometrial carcinoma is higher among women who experience inter-menstrual bleeding than among women who experience heavy menstrual bleeding [7]. The most important risk factor of EH is chronic exposure to endogenous or exogenous estrogen, which can occur in women who have yet to give birth or who are infertile; who experience earlier menarche or later menopause; who receive tamoxifen; or who experience anovulation, menopausal transition, or polycystic ovarian syndrome [8]. Obesity, diabetes, hypertension, and Lynch syndrome also increase the risk of EH [9].

EH with atypia can be treated using a total hysterectomy with or without a bilateral salpingo-oophorectomy (BSO) to eliminate the cancer risk if the woman does not wish to bear children. If she does wish to preserve fertility or she cannot tolerate a hysterectomy, she can be given oral or local progestins, aromatase inhibitors, or gonadotropin-releasing hormone agonists [10,11].

EH without atypia can be managed through watchful waiting [12], or it can be treated using oral progestins, aromatase inhibitors, and letrozole (LET) [1], or a levonorgestrel-releasing intrauterine system (LNG-IUS). The LNG-IUS may be the better choice for many patients, because of its efficacy and generally tolerable side effects [13], which include pelvic pain, breast tenderness, ovarian cysts, weight gain, and acne [14,15]. On the other hand, nearly 30% of patients experience irregular bleeding or spotting during the first three months after the involvement of the LNG-IUS. In addition, the LNG-IUS has the risk of displacement, embedment, and perforation. Patients with LNG-IUS require regular follow-up in hospital to examine the locations of intrauterine devices (IUD) by using ultrasound or other imaging examination methods, and for some patients with a large uterus cavity or irregular shape of uterine cavity, the LNG-IUS may easily migrate down to the cervical canal or even fall out of the body [16,17]. For women who are unwilling to use the LNG-IUS or who cannot tolerate its side effects, the best alternative oral medication is unclear.

Here, we performed a systematic review and network meta-analysis of the available clinical evidence to compare the efficacy of oral progestins, other oral medications, or the LNG-IUS for EH with or without atypia.

## 2. Materials and Methods

This network meta-analysis was performed in strict accordance with the Preferred Reporting Items for Systematic Reviews and Meta-Analyses (PRISMA) statement. The study protocol was registered in PROSPERO (CRD42022345837).

### 2.1. Search Strategy

The following electronic databases were searched: PubMed, EMBASE, the Cochrane Library, and clinicaltrials.gov. We searched all databases from their respective inception to 17 May 2022. The search algorithm was based on the key terms: (endometrial hyperplasia) AND ((levonorgestrel) OR (medroxyprogesterone acetate) OR (megestrol acetate) OR (progesterone) OR (norethisterone) OR (dydrogesterone)). Reference lists of included studies and previous systematic reviews were manually reviewed in order to identify additional studies. In cases of multiple studies reporting on the same patient population, only the largest study was included.

### 2.2. Study Eligibility

Studies were included in the present meta-analysis if they fulfilled the following criteria: (1) patients were diagnosed with endometrial hyperplasia with or without atypia; (2) patients were treated with the LNG-IUS, oral progestins (medroxyprogesterone acetate (MPA), megestrol acetate (MA), progesterone, norethisterone (NET), dydrogesterone (DGT), micronized progesterone (MP), or lynestrenol (LYN)), or oral non-progestins (metformin (MET), LET); (3) data sufficient to calculate regression rates were reported; (4) the study design was randomized controlled trial (RCT) or observational cohort study, whether prospective or retrospective.

We excluded studies if: (1) the participants were diagnosed with endometrial carcinoma; (2) the original data were not reported, such as in the case of reviews, study protocols, comments, or letters; (3) necessary data could not be obtained; (4) the studies had a single-arm cohort design; or (5) the studies were published in a language other than English.

### 2.3. Study Selection

All literature searches were screened independently by two reviewers, and any discrepancies were resolved by discussion between them or together with the corresponding author. The studies were screened for eligibility initially based on their titles and abstracts, then based on a review of the full text.

### 2.4. Quality Assessment

The quality of the RCTs was evaluated using the Cochrane Risk of Bias (RoB) assessment tool 2.0 (RoB 2.0) [18], and the risk of bias in the following domains was classified as low, high, or unclear: randomization process, deviations from intended interventions, missing outcome data, measurement of the outcome, and selection of the reported result. The quality of non-randomized studies was assessed using the Risk of Bias in Non-Randomized Studies of Interventions (ROBINS-I) tool, and the risk of bias in the following domains was classified as low, moderate, high, or critical: confounding, selection of participants, classification of interventions, deviation from intended intervention, missing data, measurement and reporting of outcomes [19]. The publication bias was assessed using Begg–Mazumdar rank correlation and funnel plots [20]. Any discrepancies during the quality assessment were resolved through discussion with the corresponding author.

### 2.5. Data Extraction and Calculations of Outcome

Two reviewers independently extracted the following data from each study: name of authors, publication year, study design, EH subtypes (with or without atypia), numbers of total patients and patients who experienced regression, and follow-up. Regression was defined as when the endometrial biopsy during follow-up was described as appearing “proliferative”, “secretory”, “inactive”, or “atrophic”, or as indicating a “progesterone effect” [9]. The regression rate was calculated as the number of patients with regression, divided by the total number of patients who received medication [13].

### 2.6. Statistical Analysis

The meta-analysis was performed using Stata 14.0 (StataCorp, College Station, TX, USA). Results associated with *p* values < 0.05 were considered significant. The rates of regression were compared in terms of the relative ratios (RRs) and 95% confidence intervals (CIs) using the random-effect and DerSimonian–Laird methods [21]. Heterogeneity was assessed based on I^2^ values and a visual analysis of forest plots. We considered I^2^ > 50% as high heterogeneity, in which case we conducted subgroup and sensitivity analyses, and drew Galbraith plot to obtain more detailed insights and to assess potential sources of heterogeneity [22]. The subgroup analyses were based on the EH subtype, country, and study design. The sensitivity analyses were performed by removing one study at a time and repeating the meta-analysis. 

A network meta-analysis was performed using Aggregate Data Drug Information System (ADDIS) 1.16.8, which uses a Bayesian approach and allows comparisons among all treatment arms of studies, including direct and indirect comparisons simultaneously [23].

## 3. Results

### 3.1. Study Selection

**Figure 1:**
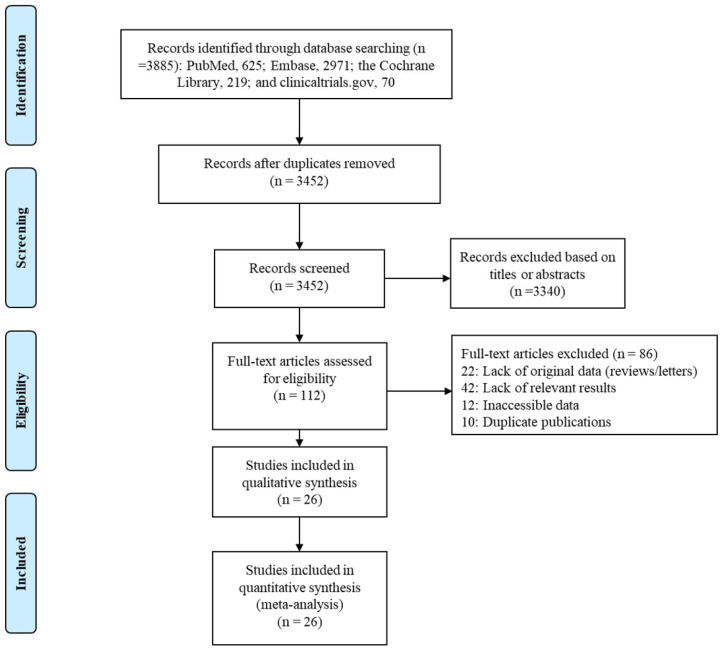
Flow diagram of study selection process.

Our search found a total of 3885 published articles—625 in PubMed, 2971 in Embase, 219 in the Cochrane Library, and 70 on clinicaltrials.gov. We removed 433 duplicate articles and excluded another 3340 based on the titles or abstracts. A full-text review of the remaining 112 articles led to the inclusion of 26 in the systematic review and network meta-analysis (Figure 1).

### 3.2. Characteristics of Included Studies

**Figure 2:**
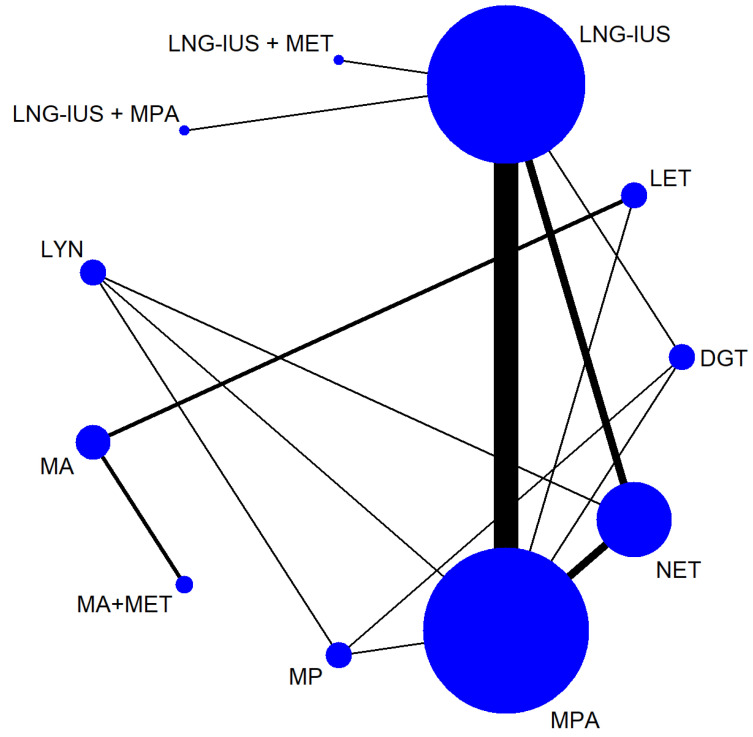
Network of comparisons among treatments for endometrial hyperplasia.

Table 1 shows the characteristics of the 26 studies, of which 5 were non-randomized and 21 were RCTs [24,25,26,27,28,29,30,31,32,33,34,35,36,37,38,39,40,41,42,43,44,45,46,47,48,49]. Altogether, the trials involved 2268 patients with EH with or without atypia. The samples in the studies ranged from 40 to 215 patients, and the studies were carried out in the following countries: Egypt (n = 6), Iran (n = 7), Turkey (n = 4), Norway (n = 4), Italy (n = 1), China (n = 1), Russia (n = 1), India (n = 1), and Pakistan (n = 1). The baseline patient characteristics were similar among the studies, allowing a network of comparisons involving 11 treatments to be analyzed (Figure 2).

In the figure, the line width is proportional to the number of trials comparing the treatments. The node size is proportional to the number of participants randomly assigned to that treatment. 

Abbreviations: DGT, dydrogestrone; LET, letrozole; LNG-IUS, levonorgestrel-releasing intrauterine system; LYN, lynestrenol; MA, megestrol acetate; MET, metformin; MP, micronized progesterone; MPA, medroxyprogesterone acetate; NET, norethisterone.

### 3.3. Quality Assessment of Included Studies

**Figure 3:**
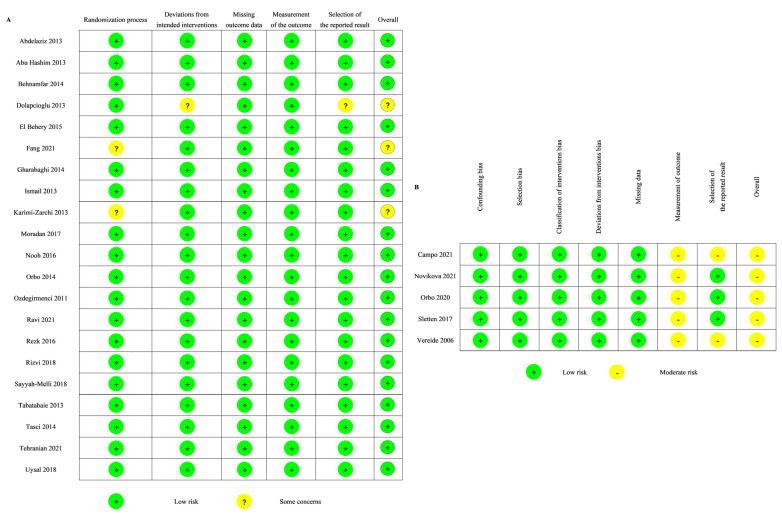
Risk of bias of the included studies: (**A**) RoB2 [24,25,26,28,29,30,31,32,33,34,35,38,39,40,41,42,43,45,46,47,48]; (**B**) ROBIN-I [27,36,37,44,49].

The quality of the RCTs was evaluated using the RoB 2.0 tool. Nearly all RCTs (18 of 21) were classified as being at low risk of bias (Figure 3A). Two of the 21 RCTs had some concerns regarding the randomization process. One of the 21 RCTs had some concerns regarding the deviations from intended intervention and the selection of the reported results.

The quality of the non-randomized studies was assessed using the ROBINS-I tool. Five non-randomized studies were classified as being at moderate risk of bias (Figure 3B). All of them were evaluated as having a moderate risk of bias in the measurement of the outcome, and two of them were also evaluated as having a moderate risk of bias in the selection of the reported result.

### 3.4. Comparisons of Regression Rates after Different Treatments

#### 3.4.1. LNG-IUS vs. MPA

**Figure 4:**
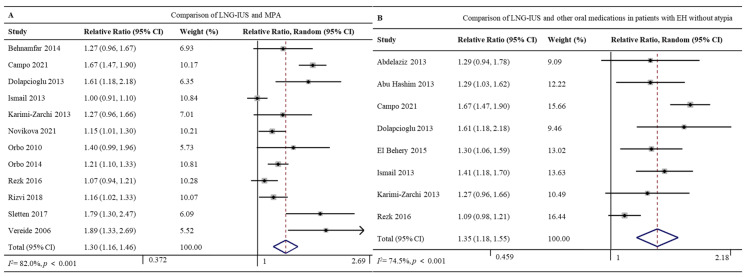
Forest plot of the meta-analysis of regression rates after treatment with (**A**) a comparison of the LNG-IUS and the MPA [26,27,28,32,33,36,37,38,41,42,44,49] or (**B**) a comparison of the LNG-IUS and other oral medications in patients with EH without atypia [24,25,27,28,29,32,33,36,41,44]. Abbreviations: RR, relative ratio; CI, confidence interval.

Twelve studies involving 1047 patients reported the regression rates for the LNG-IUS (96.7%, 408/422) and MPA (71.7%, 448/625) [26,27,28,32,33,36,37,38,41,42,44,49]. The LNG-IUS was associated with a significantly higher regression rate (RR 1.30, 95% CI 1.16–1.46, *p* < 0.001; I^2^ = 82.0%; Figure 4A). Given the high heterogeneity of the pooled data, we did subgroup analyses and sensitivity analyses but failed to uncover clear differences among subgroups. The Galbraith plot showed four studies might be the potential sources of heterogeneity [27,32,44,49] (Supplementary Figure S1).

#### 3.4.2. LNG-IUS vs. Oral Medications in Patients with EH without Atypia

Eight studies involving 882 patients with EH without atypia reported regression rates for the LNG-IUS (88.8%, 277/312) and oral medications (MPA, NET, or DGT) (66.5%, 379/570) [24,25,27,28,29,32,33,41]. The LNG-IUS was associated with a significantly higher regression rate (RR 1.35, 95% CI 1.18–1.55, *p* < 0.001; I^2^ = 74.5%; Figure 4B). Given the high heterogeneity of the pooled data, we did subgroup analyses and sensitivity analyses but failed to uncover clear differences among subgroups. The Galbraith plot showed two studies might be the potential sources of heterogeneity [27,41] (Supplementary Figure S2).

#### 3.4.3. LNG-IUS vs. NET

**Figure 5:**
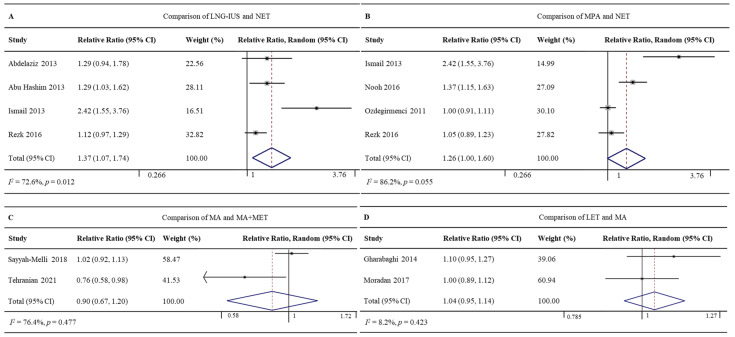
Forest plot of the meta-analysis of regression rates in (**A**) a comparison of LNG-IUS and the NET [24,25,32,41], (**B**) a comparison of MPA and NET [32,35,39,41], (**C**) a comparison of MA and MA + MET [43,47], or (**D**) a comparison of LET and MA [31,34].

Four RCTs involving 357 patients reported regression rates for the LNG-IUS (86.5%, 154/178) and NET (64.2%, 115/179) [24,25,32,41]. The LNG-IUS was associated with a significantly higher regression rate (RR 1.37, 95% CI 1.07–1.74, *p* = 0.012; I^2^ = 72.6%; Figure 5A). Given the high heterogeneity of the pooled data, we did subgroup analyses but failed to uncover clear differences among subgroups. Sensitivity analyses showed one study might be the potential source of heterogeneity [32]. Excluding this study led to the same result as the full meta-analysis, but with lower heterogeneity (RR 1.18, 95%CI 1.06–1.32, *p* = 0.003; I^2^ = 0.0%; Supplementary Figure S3).

#### 3.4.4. MPA vs. NET

Four RCTs involving 363 patients reported regression rates for MPA (92.3%, 169/183) and NET (71.7%, 129/180) [32,35,39,41]. The rates did not differ significantly between the two groups (RR 1.26, 95% CI 1.00–1.60, *p* = 0.055; I^2^ = 86.2%; Figure 5B). Given the high heterogeneity of the pooled data, we did subgroup analyses and sensitivity analyses but failed to uncover clear differences among subgroups. The Galbraith plot showed four studies might be the potential sources of heterogeneity [32,35] (Supplementary Figure S4).

#### 3.4.5. MA vs. MA+MET

Two RCTs involving 140 patients reported regression rates for MA (85.1%, 57/67) and MA+MET (93.2%, 68/73) [43,47]. The rates did not differ significantly between the two groups (RR 0.90, 95% CI 0.67–1.20, *p* = 0.477; I^2^ = 76.4%; Figure 5C).

#### 3.4.6. LET vs. MA

Two RCTs involving 142 patients reported regression rates for LET (94.4%, 67/71) and MA (88.7%, 63/71) [31,34]. The rates did not differ significantly between the two groups (RR 1.04, 95% CI 0.95–1.14, *p* = 0.423; I^2^ = 8.2%; Figure 5D).

### 3.5. Network Meta-Analysis of Regression Rates

**Figure 6:**
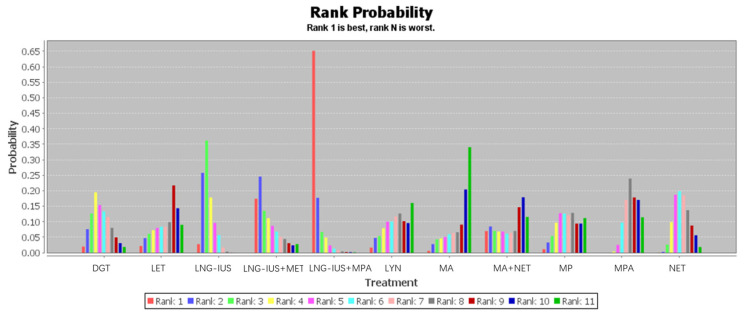
Ranking of the 11 treatments for endometrial hyperplasia based on regression rate. Rank 1 indicates the best regression rate and rank 11 the worst.

Twenty-six studies involving 2268 patients contributed to our network meta-analysis of regression rates after treatment with one of 11 regimens (Table 2 and Figure 6). The rank probability of regression across all patients showed the following trend: LNG-IUS+MPA > LNG-IUS+MET > LNG-IUS > DGT > NET > MP > MPA > LYN > LET > MA+MET > MA. The LNG-IUS was ranked higher than any of the oral medications on their own, and combining the LNG-IUS with oral MPA or MET shifted the LNG-IUS to the two highest rank positions. Among the oral medications on their own, DGT was ranked at the top.

### 3.6. Publication Bias

**Figure 7:**
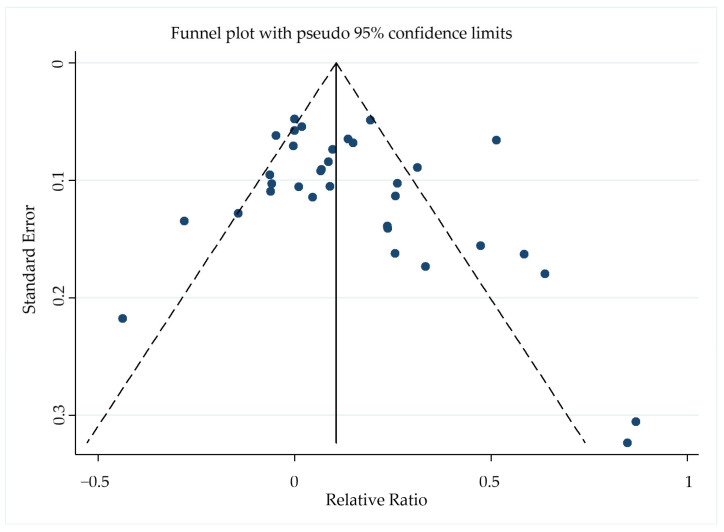
Funnel plot of the 26 included studies with pseudo−95% confidence limits based on the relative ratio for regression rates.

The Begg–Mazumdar rank correlation test showed no evidence of publication bias in the meta-analysis of regression rates (*p* = 0.152), and the funnel plot was symmetrical (Figure 7).

## 4. Discussion

In this network meta-analysis, we evaluated the effectiveness of different medications in the treatment of EH with or without atypia. Among the oral medications used individually, DGT may be superior to other progestins or non-progestins. Among any of the medications used individually, the LNG-IUS seems to be associated with a higher regression rate than oral progestins or non-progestins. Consistently, two previous studies suggested that the LNG-IUS was more effective than other oral progestins [10,50]. The present review substantially extends those findings by examining a much larger sample and by comparing the LNG-IUS and non-progestins.

Among patients with EH without atypia, the present meta-analysis showed that the LNG-IUS was associated with a significantly higher regression rate than other treatments, similar to another meta-review involving fewer studies, only a few of which were also included in the present analysis [50]. In contrast, another meta-analysis, which involved only single-arm studies that conducted purely indirect comparisons of the LNG-IUS and oral medications, found no significant difference in regression rates between oral medications and the LNG-IUS [51]. Our network meta-analysis suggests that the combinations of the LNG-IUS with MPA or MET are superior to the LNG-IUS or MPA on their own. Our work supports the use of the LNG-IUS+MPA for young women with early-stage endometrial carcinoma who want to preserve their fertility [52,53]. Our work also supports previous studies that concluded that adding MET or LET can increase the efficacy of the LNG-IUS or oral progestins [45,54]. Non-progestins may increase the efficacy of progestins on their own by upregulating the progesterone receptor in the endometrium [55].

Our findings should be interpreted with caution because of its limitations. First, the samples were quite small for some treatment regimens, especially the LNG-IUS+MPA (27 of 2268 patients), LET (92), LYN (55), LNG-IUS+MET (25), MA+MET (73), and MP (61) treatments. Second, our meta-analysis pooled data from RCTs and non-randomized studies, which differed substantially in size and potentially in heterogeneity. Indeed, the included studies differed markedly in their medication dose and the usage method of drugs, as well as the duration of follow-up. Patient compliance also plays an important role in the treatment of EH, while women treated with the LNG-IUS need to go to the hospital regularly to make sure the location of the IUD and women treated with oral medication need to take their medicine according to their physician’s orders. Third, our study did not compare the safety of the different treatments. For patients receiving the LNG-IUS, the IUD may migrate to any place other than the uterine cavity. Patients with EH who take oral progestins for a long time may have bloating, nausea, headaches, and mood swings or even depression, and the use of oral progestins may lead to liver function damage. Our study also did not compare the curative effect and safety between oral progestins and non-progestins as a result of lacking relevant data. More studies are needed. Fourth, the included studies did not uniformly report sufficient data for us to compare the treatments in terms of other clinically important outcomes, such as menstrual blood loss or other symptomatic improvements. The same was true for patient characteristics such as obesity or the presence of diabetes mellitus, whose potential influence we could not assess in the subgroup analyses [50,56]. We thought that the same characteristics as the risk factors of EH were related to the treatment effect. Fifth, most of the patients in the included studies were younger than 50 years old, and there were no suitable studies about the therapies of EH with the women aged around 50–60, even though these women are more susceptible to EH [5]. Although we did not get enough information about the race of the women included, we did find that the LNG-IUS is more used in economically advanced countries based on the countries of each study. Sixth, the treatment of patients with EH is a long-term treatment, and our study did not compare the recurrence rate and resistant rate of different therapies. Seventh, we observed the high heterogeneity in regression rate between LNG-IUS and MPA groups, LNG-IUS and oral medications groups, LNG-IUS and NET groups, MA and MA+MET groups, and we found the potential sources by using subgroup analyses, sensitivity analyses and Galbraith plots. After removing those studies, we obtained similar results, this suggests that even our more heterogeneous meta-analyses are reliable. We also observed the high heterogeneity in regression rate between MA and MA+MET groups, and we thought the reason might be there were only two studies and too few patients. More research is needed to support whether MET can increase the efficacy of MA. Lastly, we may have introduced bias by including only English-language studies, yet our analysis suggests a low risk of such bias.

Nowadays, people are beginning to pay more attention to their own health, and when there is a sign something’s wrong, such as abnormal uterine bleeding, many people will go to hospitals for professional instruction and treatment. Additionally, with the development of medical technology, the diagnosis rate of EH has been increasing gradually in recent years, especially in perimenopausal women and postmenopausal women. Some studies have suggested that calculating the endometrial thickness of postmenopausal women using an endovaginal color dopple has similar diagnostic accuracy compared with a histopathologic diagnosis [57]. The diagnosis methods of EH include dilatation and curettage (D&C) and an endometrial biopsy with a hysteroscopy [58,59]. The treatment of EH should be individualized according to age, fertility demands, personal conditions, and other factors. For postmenopausal women with atypical EH, hysterectomy is the most suitable treatment. For premenopausal women with EH who want to preserve their fertility, the LNG-IUS and oral medication are more suitable [8]. Our study has compared the therapeutic effect to EH with or without atypia between the LNG-IUS and oral medication, and we hope the outcomes of our study can help clinicians find the most appropriate treatment for these patients.

In spite of these limitations, the present work appears to be the first network meta-analysis suggesting that the LNG-IUS may be the most appropriate choice for patients with EH with or without atypia, and that combining the LNG-IUS with MPA or MET may further increase the regression rate. Our work also suggests that among the oral medications, DGT may be the most appropriate choice for women who are unwilling to use the LNG-IUS or who cannot tolerate its side effects.

## Figures and Tables

**Table 1:** Characteristics of included studies of patients with endometrial hyperplasia.

Study	Year	Country	Study Design	Endometrial Hyperplasia Type(s) (with or without Atypia)	Mean Age (Years)	Total Sample	Treatment Arms (n/N)			Follow-Up (Months)
Abdelaziz [24]	2013	Egypt	Randomized controlled trial	Without	41.7	84	LNG-IUS 31/42	NET 24/42		Range, 7–24
Abu Hashim [25]	2013	Egypt	Randomized controlled trial	Without	44.8	113	LNG-IUS 47/56	NET 37/57		12
Behnamfar [26]	2014	Iran	Randomized controlled trial	Both	38.4	55	LNG-IUS 25/28	MPA 19/27		3
Campo [27]	2021	Italy	Comparative Study	Without	38.3	215	LNG-IUS 28/28	MPA 110/187		20
Dolapcioglu [28]	2013	Turkey	Randomized controlled trial	Without	<50	52	LNG-IUS 26/26	MPA 16/26		24
El Behery [29]	2015	Egypt	Randomized controlled trial	Without	41.5	138	LNG-IUS 50/60	DGT 50/78		12
Fang [30]	2021	China	Randomized controlled trial	With	<50	47	LNG-IUS 11/20	LNG-IUS+MPA 23/27		6
Gharabaghi [31]	2014	Iran	Randomized controlled trial	Without	50.3	92	LET 43/46	MA 39/46		4
Ismail [32]	2013	Egypt	Randomized controlled trial	Without	44.0	90	LNG-IUS 29/30	MPA 29/30	NET 12/30	6
Karimi-Zarchi [33]	2013	Iran	Randomized controlled trial	Without	<50	40	LNG-IUS 19/20	MPA 15/20		3
Moradan [34]	2017	Iran	Randomized controlled trial	Without	46.2	50	LET 24/25	MA 24/25		12
Nooh [35]	2016	Egypt	Randomized controlled trial	Without	38.5	146	MPA 67/73	NET 49/73		6
Novikova [36]	2021	Russia	Comparative Study	With	<50	84	LNG-IUS 45/45	MPA 34/39		Range, 3–136
Orbo [37]	2010	Norway	Comparative Study	Both	49.3	41	LNG-IUS 25/25	MPA 11/16		Range, 59–106
Orbo [38]	2014	Norway	Randomized controlled trial	Both	Not reported	153	LNG-IUS 53/53	MPA 82/100		6
Ozdegirmenci [39]	2011	Turkey	Randomized controlled trial	Without	45.0	82	MPA 29/30	NET 26/27	LYN 24/25	6
Ravi [40]	2021	India	Randomized controlled trial	Without	44.5	49	LNG-IUS 22/24	LNG-IUS+MET 23/25		6
Rezk [41]	2016	Egypt	Randomized controlled trial	Without	44.9	150	LNG-IUS 47/50	MPA 44/50	NET 42/50	24
Rizvi [42]	2018	Pakistan	Randomized controlled trial	Both	Not reported	140	LNG-IUS 65/70	MPA 56/70		6
Sayyah-Melli [43]	2018	Iran	Randomized controlled trial	Both	44.9	84	MA 38/40	MA+MET 41/44		3
Sletten [44]	2017	Norway	Comparative Study	Both	<50	57	LNG-IUS 26/26	MPA 17/31		Mean, 155.4
Tabatabaie [45]	2013	Iran	Randomized controlled trial	Without	<50	41	MPA 20/20	LET 21/21		3
Tasci [46]	2014	Turkey	Randomized controlled trial	Without	45.5	60	LYN 29/30	MP 29/30		3
Tehranian [47]	2021	Iran	Randomized controlled trial	Without	44.0	56	MA 19/27	MA+MET 27/29		3
Uysal [48]	2018	Turkey	Randomized controlled trial	Without	45.2	99	MPA 31/35	DGT 32/33	MP 29/31	6
Vereide [49]	2006	Norway	Comparative Study	Both	50	50	LNG-IUS 21/21	MPA 15/29		3
Total						2286	LNG-IUS 570/624, MPA 595/783, NET 190/279, DGT 82/111, LNG-IUS+MPA 23/27, MA 120/138, LET 88/92, LYN 53/55, LNG-IUS+MET 23/25, MA+MET 68/73, MP 58/61			

**Table 2:** Network meta-analysis of regression rates in patients with endometrial hyperplasia after the indicated treatments.

DGT	0.30 (0.00, 36.72)	2.45 (0.30, 17.68)	2.56 (0.05, 119.04)	12.78 (0.43, 391.25)	0.40 (0.01, 13.38)	0.15 (0.00, 31.05)	0.34 (0.00, 119.42)	0.47 (0.03, 8.05)	0.28 (0.03, 2.01)	0.56 (0.05, 4.85)
	LET	8.00 (0.09, 745.95)	8.66 (0.03, 2223.49)	44.39 (0.22, 7980.75)	1.31 (0.01, 295.94)	0.49 (0.05, 4.82)	1.13 (0.05, 25.70)	1.58 (0.01, 239.64)	0.89 (0.01, 74.38)	1.82 (0.02, 179.55)
		LNG-IUS	1.04 (0.04, 27.80)	5.24 (0.36, 86.74)	0.16 (0.01, 3.56)	0.06 (0.00, 8.91)	0.14 (0.00, 33.47)	0.19 (0.01, 2.74)	0.11 (0.05, 0.25)	0.23 (0.07, 0.70)
			LNG-IUS+MET	5.12 (0.07, 359.59)	0.16 (0.00, 12.16)	0.06 (0.00, 23.78)	0.13 (0.00, 75.34)	0.19 (0.00, 11.33)	0.11 (0.00, 2.97)	0.22 (0.01, 6.46)
				LNG-IUS+MPA	0.03 (0.00, 1.83)	0.01 (0.00, 3.57)	0.03 (0.00, 12.94)	0.04 (0.00, 1.55)	0.02 (0.00, 0.37)	0.04 (0.00, 0.85)
					LYN	0.38 (0.00, 115.36)	0.83 (0.00, 411.90)	1.18 (0.05, 25.17)	0.70 (0.03, 11.39)	1.43 (0.06, 26.01)
						MA	2.34 (0.29, 18.93)	3.22 (0.01, 802.09)	1.86 (0.01, 253.03)	3.76 (0.02, 630.75)
							MA+MET	1.41 (0.00, 520.71)	0.79 (0.00, 173.19)	1.61 (0.01, 394.28)
								MP	0.60 (0.04, 7.31)	1.20 (0.07, 16.72)
									MPA	1.99 (0.64, 6.65)
										NET

The values in each cell are the relative ratio (and associated 95% confidence interval) of regression rates after the indicated treatment. Each box represents a comparison of the row-defining treatment versus the column-defining treatment. Odds ratios of more than 1 favor the column-defining treatment and odds ratios of less than 1 favor the row-defining treatment.

## Data Availability

The original contributions presented in the study are included in the article. Further inquiries can be directed to the corresponding author.

## Supplementary Materials

The following are available online at https://www.mdpi.com/article/10.3390/jcm12082980/s1, Figure S1: Galbraith radial plot depicting sources of heterogeneity in regression rates between LNG-IUS and MPA group [26,27,28,32,33,36,37,38,41,42,44,49]; Figure S2: Galbraith radial plot depicting sources of heterogeneity in regression rates between LNG-IUS and oral medications in patients with EH without Atypia [24,25,27,28,29,32,33,41]; Figure S3: Forest plot of the meta-analysis of regression rates in LNG-IUS and NET groups after removal of one study [32] potentially contributing substantially to heterogeneity in the pooled data [24,25,41]; Figure S4: Galbraith radial plot depicting sources of heterogeneity in regression rates between MPA and NET groups [32,35,39,41].
