# Supplementary material for: Efficacy of Oral Medications or Intrauterine Device-Delivered Progestin in Patients with Endometrial Hyperplasia with or without Atypia: A Network Meta-Analysis
Source: J Clin Med. 2023 Apr 19;12(8):2980. doi: 10.3390/jcm12082980 (PMC10143726; doi:10.3390/jcm12082980)
Supplement: Supplementary file 1 [file jcm-12-02980-s001.zip › jcm-2215586-supplementary.pdf]

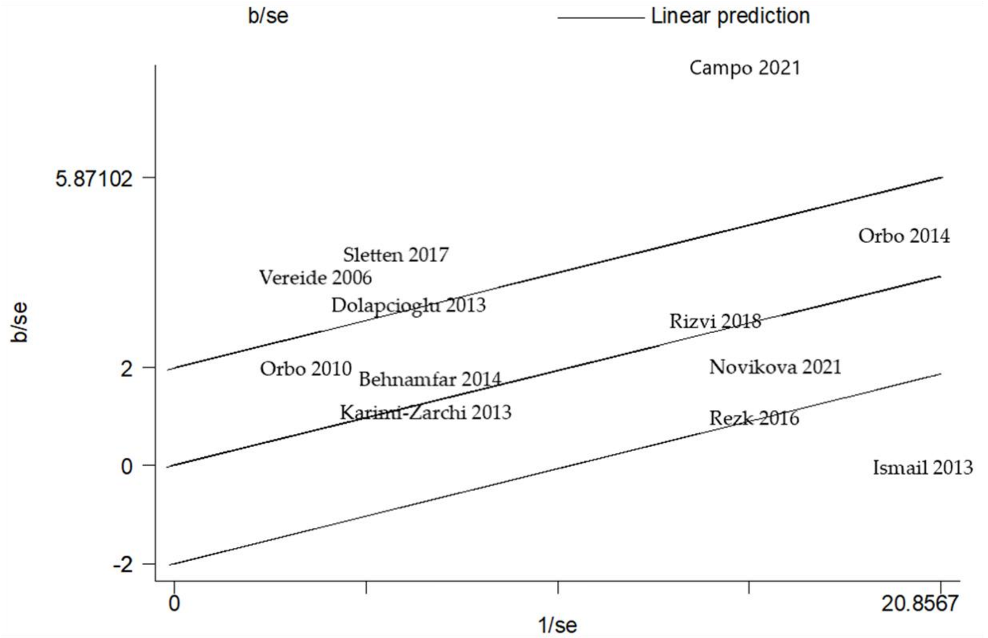

Numbers in brackets [ ] are reference numbers of included studies.
Y axis: RR /standard error of RR; X axis: 1/standard error of RR.

Supplementary Figure S1. Galbraith radial plot depicting sources of heterogeneity in regression rates between LNG-IUS and MPA groups [26–28,32,33,36–38,41,42,44,49].
Abbreviations: LNG-IUS, levonorgestrel-releasing intrauterine system; MPA, medroxyprogesterone acetate; RR, relative ratio.

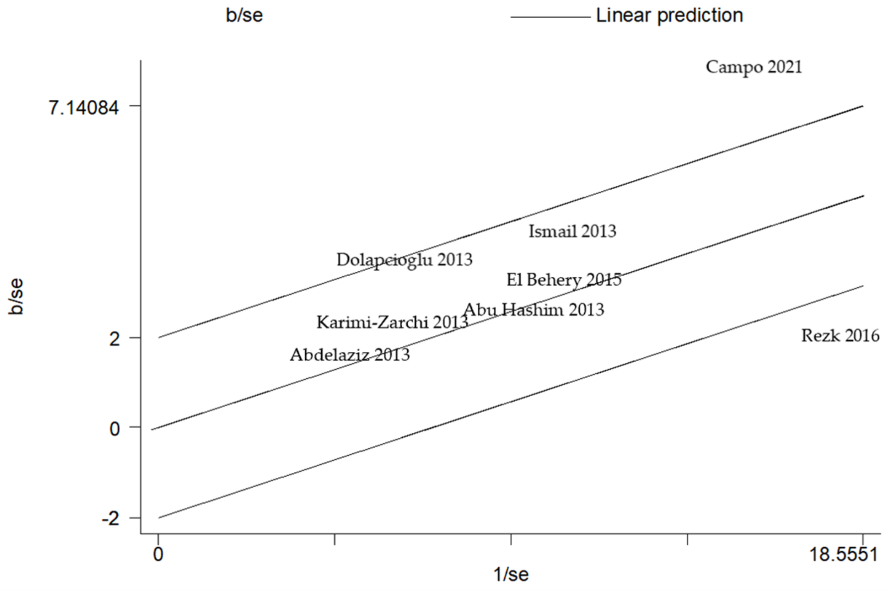

Numbers in brackets [ ] are reference numbers of included studies.
Y axis: RR /standard error of RR; X axis: 1/standard error of RR.

Supplementary Figure S2. Galbraith radial plot depicting sources of heterogeneity in regression rates between LNG-IUS and oral medications in patients with EH without Atypia [24,25,27–29,32,33,41].
Abbreviations: LNG-IUS, levonorgestrel-releasing intrauterine system; RR, relative ratio.

Study	Relative Ratio (95% CI)	Weight (%)	Relative Ratio, Random (95% CI)
Abdelaziz 2013	1.29 (0.94, 1.78)	12.18	
Abu Hashim 2013	1.29 (1.03, 1.62)	24.83	
Rezk 2016	1.12 (0.97, 1.29)	62.99	
Total (95% CI)	1.18 (1.06, 1.32)	100.00	
$I^2 = 0.0\%$, $p = 0.003$			

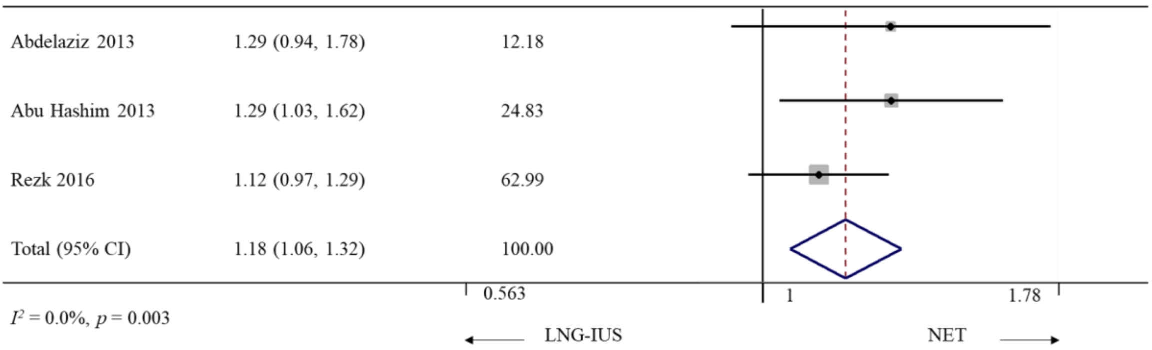

Supplementary Figure S3. Forest plot of the meta-analysis of regression rates in LNG-IUS and NET groups after removal of one study [32] potentially contributing substantially to heterogeneity in the pooled data [24–25,41].
Abbreviations: LNG-IUS, levonorgestrel-releasing intrauterine system; NET, norethisterone; RR, relative ratio; CI, confidence interval.

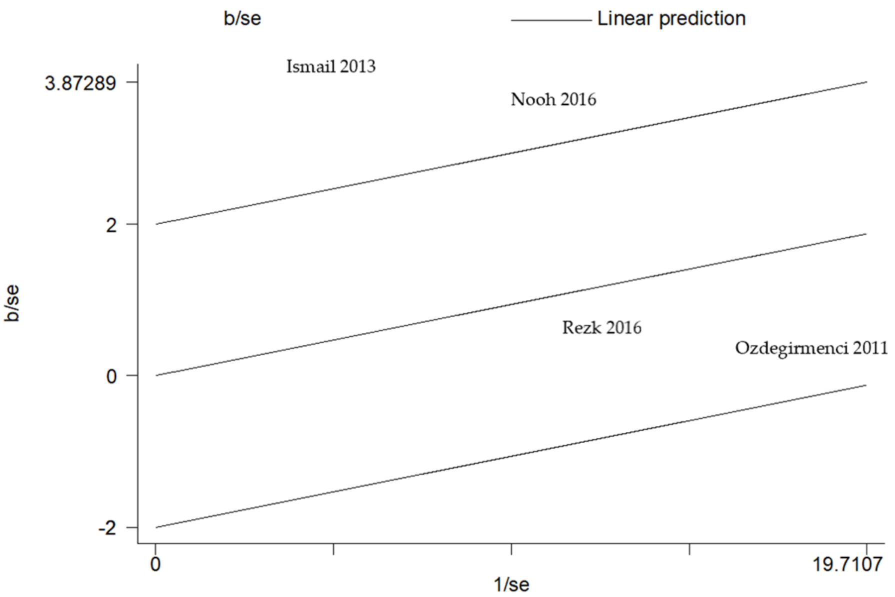

Numbers in brackets [ ] are reference numbers of included studies.
Y axis: RR /standard error of RR; X axis: 1/standard error of RR.

Supplementary Figure S4. Galbraith radial plot depicting sources of heterogeneity in regression rates between MPA and NET groups [32,35,39,41].
Abbreviations: MPA, medroxyprogesterone acetate; NET, norethisterone; RR, relative ratio.
